# “I don’t think of it as a shelter. I say I’m going home”: a qualitative evaluation of a low-threshold shelter for women who use drugs

**DOI:** 10.1186/s12954-024-00930-1

**Published:** 2024-02-19

**Authors:** Corinne A. Beaugard, Fay Khudairi, Oluwatoyin Yesufu, Andrea Farina, Jordana Laks

**Affiliations:** 1 School of Social Work, Boston University, 264 Bay State Road, Boston, MA 02215 USA; 2 Boston Health Care for the Homeless Program, 780 Albany Street, Boston, MA 02118 USA; 3 St. Francis House, 39 Boylston St, Boston, MA 02116 USA; 4 Chobanian and Avedisian School of Medicine, Department of Internal Medicine, Boston University, 801 Massachusetts Ave 6th Floor, Boston, MA 02119 USA; 5 Present Address: 801 Massachusetts Avenue, Crosstown Building 4th Floor, Suite 400, Boston, MA 02180 USA

**Keywords:** Low-threshold shelter, Sweeps, Harm reduction, Encampments, Homelessness, Addiction, Substance use disorders

## Abstract

**Background:**

In 2021–2022, encampments in a downtown Boston neighborhood reached record heights, increasing the visibility of drug use and homelessness in the city. In response, the city planned a “sweep” (i.e., eradication of encampments) and requested support from social services and medical providers to pilot low-threshold shelters. Low-threshold shelters reduce barriers to staying in traditional congregate shelters with more flexible regulations, longer-term bed assignments, and secured storage for contraband (e.g., drugs, weapons) instead of forced disposal. One homeless service provider opened a harm reduction-focused shelter for women who use drugs. This report describes the low-threshold shelter design and program evaluation.

**Methods:**

This program evaluation had two primary aims: (1) to examine guests’ beliefs about shelter policies and practices; and (2) to understand the staff’s experiences working in a low-threshold model. We conducted semi-structured qualitative interviews with 16 guests and 12 staff members during the summer 2022. Interviews were thematically analyzed.

**Results:**

Guests expressed overwhelming approval for the shelter’s policies, which they stated supported their autonomy, dignity, and safety. They emphasized the staff’s willingness to build relationships, thus demonstrating true commitment to the guests. Guests highlighted the value of daytime access to the shelter, as it granted them autonomy over their time, reduced their substance use, and helped them build relationships with staff and other guests. The co-directors and staff designed the shelter quickly and without US models for reference; they turned to international literature, local harm reduction health care providers, and women living in encampments for guidance on the shelter policies. The staff were passionate and committed to the health and stability of the guests. Most staff found value in the low-threshold model, though some were challenged by it, believing it enabled drug use and did not require the guests to “get better.”

**Conclusions:**

This evaluation indicates the value of low-threshold, harm reduction shelters as alternatives to traditional models. While these shelters do not mitigate the need for overarching housing reform, they are important measures to meet the needs of women experiencing unsheltered homelessness who face intersectional oppression.

**Supplementary Information:**

The online version contains supplementary material available at 10.1186/s12954-024-00930-1.

## Background

Homelessness continues to rise in the United States, reaching a record-high of 653,100 people (20 out of every 10,000 people) according to the 2023 Point-in-Time count [1]. Homelessness is associated with premature mortality and high rates of chronic medical conditions (e.g., anemia, viral hepatitis, and chronic obstructive lung disease), psychiatric disorders (e.g., mood, anxiety, and psychotic disorders), and substance use disorders (SUDs) [2–6]. Standardized mortality rates for homeless populations are 8–16 times higher than comparison groups, with cancer, heart disease, and complications from substance use as the leading causes of death [2]. Substance use disorders are at least twice as prevalent among adults experiencing homelessness compared to the general population, with rates as high as 30–50% [7–9]. People with SUDs experiencing homelessness suffer greater drug-related health consequences, including higher rates of overdose death, suicide, and infections, and may be more likely to engage in high-risk drug use practices (e.g., sharing supplies) [10–12]. One study in Boston, Massachusetts found that the rate of overdose deaths in the homeless population was 12 times higher than the state’s adult population from 2003 to 2017 [13].

The potential for drug-related harms is especially high in the unsheltered homeless population because of barriers to medical care and harm reduction services, limited access to hygienic environments, and severe environmental conditions [10, 14]. Nationally, the unsheltered population increased by 10% from 2010 to 2023 and, in 2023, 4 of 10 people experiencing homelessness were unsheltered [1]. Unsheltered homelessness persists in part because of the barriers associated with traditional shelters, including single-night bed assignments, curfew enforcement, strict behavioral regulations, and punitive responses to substance use [15–17]. Encampments offer an appealing alternative for some because they provide greater autonomy and community [15, 18]. While encampments are less restrictive, they expose residents to potential harms, including weather-related hazards, physical, sexual, and emotional violence, theft, and state-orchestrated displacements or “sweeps” [5, 15, 19, 20].

Women experiencing unsheltered homelessness face heightened stigma and violence due to multiple marginalized identities; they are at risk for discrimination, gender-based violence, coerced substance use, sexual exploitation, and criminalization of drug use while pregnant or parenting [5, 12, 14, 21, 22]. Rates of severe psychiatric disorders and childhood trauma are also higher in women who use drugs compared to men [4, 11]. Women-only shelters offer a safe space and opportunity to provide trauma-informed care, build community and trust, and avoid recurrent trauma sometimes experienced in mixed-gender programs [21, 23].

Low-threshold shelters are one promising short-term intervention for people experiencing unsheltered homelessness who avoid traditional shelters due to drug use, fear of losing their encampment, or other reasons [18, 24]. Low-threshold shelter models vary, but typically they allow guests to stay regardless of substance use, have flexible times to shower and eat, offer assigned beds, and provide amnesty boxes (i.e., storage for drugs, drug supplies, and weapons) [18, 25]. Low-threshold shelters in Philadelphia, Pennsylvania and Ontario, Canada have documented their approaches to harm reduction, which include minimizing long-term shelter bans, supporting mental and physical health without substance use stipulations, providing safer use supplies, and implementing overdose prevention strategies [26–28]. However, there is little research on the experiences of guests staying in low-threshold shelters or the staff who work in them.

To address this literature gap, we describe the design, implementation, and program evaluation of a low-threshold shelter for women with SUD experiencing unsheltered homelessness in Boston, MA. This evaluation was designed to: (1) learn about guests’ experiences in the shelter and their perspectives on shelter policies and practices; (2) explore the co-directors’ and staffs’ experiences designing and operating the shelter; and (3) determine alignment between guest and staff perspectives.

## Methods

### Evaluation city: Boston, Massachusetts

The 2023 Point-in-Time count identified 19,141 people experiencing homelessness in Massachusetts, 7% of whom were unsheltered (*n* = 1362); this population has grown by 27% since 2007 [1]. In the same year, 5202 people were experiencing homelessness in Boston, a 17% increase from 2022; this count included 169 unsheltered individuals, a 42% increase from 2022 [29]. In Boston, the housing crisis, contaminated drug supply, and closure of major addiction treatment and shelter programs in 2014 resulted in a concentration of individuals with SUDs living in an encampment near the intersection of Massachusetts Ave and Melnea Cass Blvd (known as “Mass and Cass”). Caps on treatment and shelter capacity in Massachusetts during the COVID-19 pandemic further strained existing demand for housing options to serve Boston’s growing encampment population. Scenes of concentrated street homelessness, public drug use, and high levels of reported physical and sexual violence drew public outcry and political attention [30, 31]. In response, the city government scheduled a sweep of the Mass and Cass encampment for January 12, 2022, and contracted local service providers to open and operate low-threshold shelters for individuals displaced by the sweep [32].

### Evaluation setting: shelter

In collaboration with the city public health department, a local homeless service provider designed and opened a low-threshold shelter to serve unaccompanied women (≥ 18 years old) who were living in the Mass and Cass encampment. Guests at this shelter primarily used fentanyl, although polysubstance use was the norm in this community, with use of cocaine, methamphetamine, alcohol, and non-prescribed sedative pills all common. Most guests injected drugs, but other routes of substance use were also common. The shelter opened shortly before the sweep in December 2021 with a capacity for 23 guests. It was located on the top floor of a building that houses the city-run, women-only emergency shelter on lower floors.

#### Staffing and training

The shelter employed eight full-time and two part-time staff members, including guest engagement specialists, contracted security staff, and a part-time contracted nurse. A neighboring community health agency staffed visiting healthcare personnel including the psychiatric nurse practitioner and harm reduction nurses. The shelter had three shifts: morning 7 AM–3 PM, afternoon 3 PM–11 PM, and overnight 11 PM–7 AM. Staff were assigned primary shifts, but many worked additional shifts as well. At least two staff members plus security were present in the shelter 24/7. Guest engagement specialists were responsible for a range of tasks involved in maintaining shelter operations, monitoring for safety, responding to crises including overdose, and providing guests with emotional support, connection to services, and supplies (e.g., condoms, clothing). Security staff were stationed at the center administrative desk, freeing engagement specialists to do operations tasks and build relationships with the guests. Although lived experience was not a requirement of the position, many guest engagement specialists had personal or family experience with homelessness and/or SUDs.

A harm reduction specialty nurse was available during daytime hours for training, mentoring, and as-needed consultations. Overnight, a contracted nurse provided on-site medical attention and overdose response. Shelter staff connected guests to health services (e.g., medical, gynecological, and psychiatric care) from a partner community health center. Urgent medical care from a nurse or provider was available on-site during weekday evenings, with comprehensive care available at their primary clinic located adjacent to the shelter. A psychiatric nurse practitioner saw patients in the shelter every other week. A part-time housing navigator helped guests complete housing applications and facilitated placements.

The harm reduction specialist nurses trained shelter staff on SUDs, stigma, key drug effects and withdrawal syndromes, and harm reduction principles and practices, including opioid overdose response with intranasal naloxone. Staff were also trained in sedation monitoring and distinguishing between sedation that could be safely observed from overdose requiring naloxone. Shelter co-directors trained staff in a trauma-informed approach with attention to the needs of women experiencing homelessness, such as support with filing police reports after sexual assault or intimate partner violence.

#### Harm reduction policies and practices

The shelter policies and practices were modeled after recommendations made by low-threshold shelters in Canada [27, 33], insight from local harm-reductionists, and suggestions from women living in the Mass & Cass encampment. Prior to opening, shelter staff conducted outreach in the encampment to gather insight on what women wanted in a low-threshold shelter. Their recommendations included: (1) providing no barrier access to harm reduction supplies; (2) servicing guests regardless of intoxication; (3) minimizing logistical barriers to stay in the shelter; and (4) adapting policies to guests’ needs. The homeless services provider integrated information from across sources to create a setting that would offer respectful, dignifying care for the guests. They designed the shelter to feel like a home with 2–4 beds in each room, stable bed assignments with space to store personal belongings, and a comfortable, welcoming communal dining and living space. The décor was colorful, and the space was kept clean (Additional files 1: S1, Additional files 2: S2, Additional files 3: S3). Guests had access to their rooms and the kitchen, bathrooms, and common areas 24/7.

##### Flexible check-in

Guests could reserve their bed while staying out for nights at a time. This flexibility helped guests acclimate to the shelter, build trust, continue using drugs, and connect with friends and partners. Guests were expected to check in at least once daily, by phone or in person. Unless there was an approved reason for absence (e.g., hospitalization), guests who did not stay in the shelter for over 7 days lost their bed and had their items stored for retrieval.

##### Drug use and monitoring

Guests were allowed to continue using drugs; however, substance use was not permitted inside the shelter. Staff members counseled guests on safer injection practices like cleaning the skin, warming the skin, and using safer venous access sites. Staff escorted guests outside to use substances and helped them inject when needed (e.g., helped warm skin). Inside the shelter, staff supported and monitored sedated guests using continuous pulse oximetry. In cases when guests did use substances in the shelter, staff were encouraged to take a non-punitive, individualized approach. Involuntary shelter discharge was a last-resort measure used only when a guest presented imminent safety risks, not in response to drug use, intoxication, or psychiatric distress.

The shelter implemented a series of preventative measures to prevent fatal overdose. Bathrooms stalls and showers were equipped with reverse motion detectors that alerted staff if someone was inside and had not moved for two minutes. Staff also did regular rounds to ensure guests were safe and identify overdoses. Guests were required to keep their bedroom doors slightly open.

##### Amnesty boxes

Shelter staff escorted guests into and out of the shelter for safety purposes. Before entering the shelter, guests deposited drugs, drug use supplies, or weapons into their assigned “amnesty box,” a metal box that was then secured in a locker and was only accessible to staff (Additional file 4: S4). These items were returned upon request when guests exited the shelter. This system balanced community safety with individual needs to maintain access to restricted belongings. When restricted items were found in the shelter, staff secured them in the guest’s amnesty box.

##### Harm reduction supplies

Guests had 24/7, barrier-free access to safer drug use supplies (e.g., syringes, pipes, cookers, cottons, bleach) and safe disposal of supplies. Staff provided intranasal naloxone kits to guests, and naloxone was also accessible in emergency kits located in public spaces throughout the shelter. Sexual and women’s health harm reduction supplies including condoms, lubricant, and pregnancy testswere available barrier-free, as well.

### Data collection

The evaluation team, comprised of public health, medical, and social work professionals, drafted a trial interview protocol for the guests and staff interviews. The team collaborated with a current guest provided feedback the interview protocol to ensure it was appropriate, relevant, and in the spirit of the community; this guest was compensated for her work. The staff interview protocol was designed with input from the original co-directors. The semi-structured interviews asked guests and staff about their experiences living and working in the low-threshold shelter and their perspectives on shelter policies and practices. All guests staying at the shelter during the evaluation were invited to participate. The shelter staff supported recruitment by explaining the evaluation to guests, gauging interest, and providing warm handoffs to interviewers. Of the guests who were alert and in the shelter during recruitment hours, all but one guest consented to participate. Guest interviews lasted between 15 and 35 min, and participants were compensated with a $50 Target gift card.

Staff were recruited using a purposive sampling strategy, which sought to cover the range of perspectives that could vary according the position and shift [34]. Staff interviews lasted between 20 and 60 min and were conducted during their shifts. Interviews were held between July and September 2022.

### Data analysis

Interviews were recorded and transcribed for analysis. Five guest interviews were not recorded due to technical issues (*n* = 2) or because they did not wish to be audio recorded (*n* = 3). When transcripts were not available, detailed interview notes were coded. Transcripts were thematically analyzed [35] by three members of the evaluation team [CB, FK, OY] in NVivo 12 Plus. Guest transcripts were deductively coded to capture their relationships with staff and other guests, reasons for choosing this shelter, and suggestions for improvement. Staff transcripts were deductively coded for their motivation to work at shelter, beliefs about harm reduction, and perspectives on shelter policies. Next, the transcripts were inductively coded for emergent themes related to the social and emotional experiences of the guests and staff. The evaluation team members discussed the codes until a consensus was reached.

According to the Boston University Institutional Review Board guidelines, this project was not considered research as the primary purpose was to understand and improve shelter operations and experiences.

## Results

Between December 2021 and August 2022, 73 women stayed at this shelter. On average, guests stayed 62 days; stays ranged from 4 to 246 days. Sixteen women were interviewed about their experiences in the shelter, including 15 current guests and one former guest who had moved to permanent housing. Respondents ranged in age from 36 to 64 and over half were non-Hispanic White (*n* = 9, 56%), three were Hispanic (19%), two were non-Hispanic Black (13%), one was multiracial (6%), and one had unknown race/ethnicity (6%). Staff participants (*n* = 12) included the original two co-directors, supervisors (*n* = 3), guest engagement specialists (GES, *n* = 4), nursing staff (*n* = 1), and security staff (*n* = 2). Staff members were primarily women (11/12), ranged in age from 29 to 51, and reported their racial/ethnic identities as non-Hispanic White (*n* = 4), Hispanic (*n* = 2), and non-Hispanic Black (*n* = 6). Guest and staff participants were assigned pseudonyms to report results.

Four major themes emerged from the guest interviews: (1) appreciating a safe, welcoming environment; (2) benefiting from flexible, harm reduction policies; (3) building relationships with staff; and (4) providing suggestions for improvement. Three major themes emerged from the staff interviews: (1) reflecting on the low-threshold model; (2) prioritizing relationships with guests; and (3) having lived experience with homelessness and addiction. These themes are illuminated in turn below.

### Findings from guest interviews

#### Appreciating a safe, welcoming environment

Guests were satisfied with the shelter because it felt like a home, allowing them to relax and be comfortable. Amanda shared: “When I first got here, [the staff] kept saying, ‘This is your new home.’ They made me feel like this was my new home, it says home literally on my door.” They preferred the atmosphere of this shelter over traditional shelters which Ashley described as unsafe, restrictive, and impersonal: “[This shelter] actually wasn’t quite like the general shelter…. It was more of a home setting than a shelter. I don’t think of it as a shelter. I say I’m going home.” Within the safety of this shelter, Sarah felt comfortable exploring herself and her relationships: “They kind of allow us… It’s almost like finding yourself, I guess. You know what I’m saying? So, it’s like I’m finding myself and I’m able to do it somewhere safe.” Many explained that part of this shelter’s appeal was the relief from violence experienced at other shelters or on the street. Melissa stated, “It is safe here, like we don't have women beating on women…you don't have like people showing up, or ex-boyfriends or anything like that… There's not a lot of violence here at all.” Sarah explained that staying in a safe, reliable environment allowed her to care for her basic needs: “I'm safe here, I don't have to worry about where I'm gonna sleep or if I wanna shower, and I just feel comfortable. And it's hard for me to feel comfortable in places.” Safety and comfort were driven by fewer restrictions and the ability to manage time, key elements of the low-threshold model.

Notably, interviewers did not ask about and guests did not explicitly mention survival sex, defined as the exchange of sex for food, shelter, drugs, or other material need for survival [36, 37]. However, Michelle described stopping prior behaviors that she did to meet basic needs, possibly referring to survival sex or other criminalized behaviors:

[I feel] safer…that I can actually stay somewhere and sleep somewhere. So, I mean, I know that people steal [and] all that stuff. But like I don't have to do the things that I used to do in order to have a place to sleep. I don't have to do things to... take a shower or, you know, eat something.

#### Benefitting from flexible, harm reduction policies

The guests identified the most impactful practices and policies: having their basic needs met, unrestricted 24-h access to the facility, and the ability to check in with staff by phone to retain their bed. In part because guests had their basic needs met, the shelter was low-conflict environment. According to Cynthia, “When you're on the streets, there's so many people out there, it's hard to forge friendships when your very basic needs aren't met.” Melissa agreed that access to adequate resources diminished some potential for conflict, “Nobody’s squabbling over like the amount of resource. You know what I mean? There’s enough for everyone.”

The flexibility to come and go, without sacrificing their beds or risking missing curfew, allowed women freedom in their lives:

I think that's a big reason why a lot of us stay here, especially me, because the rules are flexible, but we can also have our own life out there. It doesn't say you can choose this or that.... You can live your life. They don't tell you what life to live. (Melissa)

One of the newer guests, Hazel, explained that this policy was instrumental in her willingness to stay at this shelter: “I heard it was good, it was a good place to be. And you could still handle your business, go out when you wanted to. So that right there made me feel like I could come here.”

The flexible policies and unrestricted shelter access helped some guests reduce their drug use. With access to inside space away from people using drugs, Brooke navigated her treatment and reduced use:

And I don't like so much being on the street and using. So, I've more been trying to lean towards getting sober in like a harm reduction way though. I'm not totally clean, but I started the methadone clinic and I check in [with the shelter] all the time. I don't really hang out with anybody outside of here. I'm more like stay to myself in my room and read books and whatever.

Cynthia explained her drug use decreased naturally because she was not constantly exposed to others’ use on Mass and Cass: “It’s not right there in front of you all the time.” Even when guests were not in treatment, living in the shelter facilitated reduced use.

The guests explained that amnesty boxes attracted them to the shelter. When asked why she chose to stay at this shelter, Valerie explained that the amnesty box encouraged her to try the shelter: “I was like, ‘Oh, great. I don't have to hide stuff outside and I don't have to worry about.’ You know what I mean?” Sarah agreed that amnesty boxes fostered a positive dynamic between staff and guests: “I don't feel like I'm hiding anything, I'm being honest. And I feel like they feel the same way.” She suggested that transparency helped the guests and staff trust each other and avoid suspicion or other conflict.

#### Building relationships with staff

Participants emphasized that the staff were non-judgmental and trustworthy. Rachel reflected on her dynamic with the shelter staff: “They treat you like a human being, not like a prostitute out on the street.” Cynthia agreed that the staff treated the women with respect: “I’ve been treated with the utmost respect and dignity by the staff members…it’s sort of given me this feeling of being a civilian again…When you’re homeless, you really get treated like the worst of the worst.” The guests believed the staff’s understanding of addiction and trauma facilitated this positive rapport:

Some of the staff I guess can relate to me, maybe through recovery or stuff like that…. And they don’t have to share their story or information with me, and the fact that they feel comfortable to do so, makes me more comfortable…. ‘Cause now I’m like, ‘Oh wait, she understands and she’s not gonna look down on me.’ (Sarah)

The staff’s investment in building non-judgmental, supportive relationships with guests encouraged them to share about their experiences.

#### Providing suggestions for improvement

Though many guests were empowered by the shelter’s flexible structure, others believed increased structure would serve them better. For example, Cynthia believed structure could help guests prepare for next steps: “And I wonder if there might, should be like some more expectations put up on us because we need to be expected to contribute. We need to be expected to be able to function in society.” Eliza explained that she needed structure because it helped her be accountable to herself: “If I don’t have structure, I go buck wild.” Finally, some participants who were themselves comfortable with the shelter’s rules believed they were so lax that other guests could take advantage of them.

### Findings from staff interviews

#### Reflecting on the low-threshold model

Most of the staff were new to harm reduction and the low-threshold model, which made the collaboration with harm reduction experts essential. Laura, one co-director, explained how she acclimated staff to harm reduction practices: “[Staff will] jump into, ‘they can't have this, they can't do that. They have to be kicked out.’ That's what we were used to doing, so it's very difficult to change that part and be actually low-threshold.” Despite some reservations and little previous experience with harm reduction shelters, the co-directors witnessed how this model benefitted guests and supported retention:

The person came back. They haven't been here for three days. Now, they came back one night and then they were out for another one. Now they come back for two nights and then they came back for three nights. And yes, they're using on the floor, but guess what? They're coming back. (Laura, co-director)

Anna, the other co-director, shared Laura’s enthusiasm for the shelter policies’ positive impact on the guests. She observed that women decreased their substance use as they remained in the shelter:

I just think [the women decreased their substance use] because they are starting to see what's available to them, because they're getting some sober time. And so, I think they're starting to slow down a little bit, because it's like, ‘Oh, my God, this could be happening. We have a safe place to stay, why are we even doing this?’

The shelter was not designed with any addiction treatment or substance-related requirements. The co-directors hypothesized that increased social support, including relationships with the co-directors, and access to shelter 24/7 led to guests’ naturally decreasing their use.

Most of the interviewed staff learned about harm reduction on the job, and they reported a range of comfort implementing these practices. One of the security staff described a growing appreciation for harm reduction:

[The amnesty boxes] kind of made sense because it's almost like they'll stay out there just to look for whatever it is and then lose it, or somebody might rob them. And then they're like, ‘Well, I'm getting sick, so I need to go find something else.’ Whereas here…no one's touching [their drugs or supplies]. (Jessica, security, morning)

While staff acknowledged harm reduction’s pragmatism, many expressed ambivalence with this approach:

Harm reduction is good, and is bad because it's somewhat like babying them... It's kind of enabling... but still you're reaching—you’re meeting them where they're at. Now you'll be able to meet them where they're at and you get to communicate with them. You get to talk to them. Now you get to bring them up another level. (Richard, supervisor, overnight)

Other staff were more critical about the shelter’s harm reduction policies. One guest engagement specialist (GES) described her reservations:

I don’t like the fact that they can come in under the influence. And I understand why it is the way it is. ‘Cause there needs to be a window where they can come in first, feel safe, then maybe think about getting better. But there’s nobody saying get better. (Maggie, GES, morning/ afternoon)

The staff wanted the guests to “get better” and often discussed their desire that the guests engage in a recovery process and decrease or stop their drug use.

#### Prioritizing relationships with guests

Staff wanted to build relationships with guests and invested in individual relationships. Mary (GES, morning) said, “I have learned what… words to use when speaking with them and build a trust[ing] relationship slowly with them, so, they can let you in their circle.” Connection with guests and attunement to their needs allowed staff to be effective during high-stress scenarios: “[I] made a point to get to know the girl, each of the guests on a certain level. So that I can understand them, so that when they are highly intoxicated, I know how to talk them through things” (Cara, GES, all shifts).

Commitment to their work was challenging at times–as staff often took work home with them. Rebecca (GES, afternoon) explained:

Leaving work at work is important. And I try to do that as much as possible and not take it home so that I could take care of myself and be at the best health space, mental space, emotional space for my clients.

Working with guests navigating challenging life experiences—some of which they had personally experienced—was difficult. Alexa (supervisor, afternoon) explained that it was not possible to establish rigid boundaries between her personal and professional life:

They tell you, ‘Don't take your work at home,’ but who doesn't do that? But it does burn you out…I love it, I enjoy what I do. Just like anywhere, it has its moments where I'm just... You're like, ‘I can't handle it today.’ But, I think what helps is having strong communication and having a strong team.

Staff recognized that “taking their work home” was stressful but were motivated by commitment to the guests’ well-being.

#### Having lived experiences with homelessness and addiction

Most staff had personal and/or family history with addiction or homelessness and explained that this motivated them to work at the shelter:

I grew up around addicts. Summary - mother, father, two uncles, and an aunt. All addicts. So, it's like second nature. And that's kind of why I'm in the field, to keep my own life in line, keep in check. So yeah. It's not hard [to do this work]. (Maggie, GES, morning/ afternoon)

In some cases, staff saw their work at the shelter as part of their effort to do service and maintain their own recovery:

[I am] so grateful to still be living that I owe it, not to myself, but I owe it to them as well. I'm a living story of I could have been dead. So, I'm very motivated. It also keeps me sober one more day, one more hour. (Mary, GES, morning)

Although personal experience with addiction motivated staff to work at the shelter, it also made it difficult for some who wished to incorporate elements of addiction treatment into the shelter out of a desire to see guests “get better.”

## Discussion

This qualitative evaluation of guest and staff experiences demonstrates the important role that low-threshold shelters play in the ecosystem of homeless services. The guests’ average length of stay (62 days) speaks to the success of the model in retaining guests, making it a viable short-term option for women with SUD experiencing homelessness. The guest and staff interviews shed light on how the policies and practices that prioritized women’s safety, dignity, and autonomy fostered an environment that was appealing for women who did not want to stay in traditional shelters.

This study’s findings illuminate how low-threshold shelters mitigate some of the known issues with traditional shelters and encampments. Adults experiencing homelessness may avoid shelters because encampments provide a better opportunity to cultivate community and a sense of home (e.g., more control over personal belongings) and permit greater autonomy [15, 38]. While encampments offer some benefits, they are also associated with physical and sexual violence, especially for women. Previous research on the Mass and Cass encampment found that women reported high rates of survival sex and sexual assaults [39, 40]. Consistent with literature on homeless individuals’ experiences of safety, participants in this study felt safer in this shelter than in encampments or other local shelters, reporting freedom from coercion and less interpersonal conflict when their basic material needs were met [38, 41]. This safety was cultivated by the gender-specific setting, the staff’s ability to support women with complex mental health and trauma histories, and the low-conflict, nurturing environment [11, 41, 42]. Importantly, the shelter provided a single-gender space for women facing intersectional stigma from their gender, substance use, and street homelessness.

Beyond the safety of shelter, this setting facilitated support for intimate partner violence, sexual assault, and psychiatric concerns. Single-gender settings that integrate services dedicated to women have been associated increased service engagement in both harm reduction centers [43–45] and homeless service programs [46, 47], mitigating gendered barriers to accessing these services [11, 48]. Future models should consider integrating additional gender-informed features in low-threshold shelters, such a sexual and reproductive care, support with navigating the child welfare system, and trauma-focused mutual aid groups.

The shelter’s access policies—both unrestricted daytime access and the ability to spend nights away—fostered guests’ ability to maintain relationships with people inside and outside of the shelter. This freedom was essential in retaining guests at the shelter. Since each night spent inside is associated with health benefits, this policy was an effective way to support the social, psychological, and physical health of this population [42]. Spending nights away from the shelter allowed guests to see partners, friends and family outside of the shelter, buffering against loneliness and isolation often experienced in homelessness. The check-in policy also allowed guests to acclimate to the shelter at their own pace. Research suggests people experiencing homelessness can struggle to navigate change and new environments; offering guests the opportunity to transition at their own pace was an effective harm reduction strategy [49].

This shelter’s approach to drug use and some of the guests’ descriptions of their substance use while at the shelter (e.g., reduced use due to daytime shelter access) demonstrates the risk environment framework [12, 50]. This framework explains that drug use is exacerbated by physical environmental risk factors, including structural inequities (e.g., criminalization of drug use and homelessness, commodification of healthcare) and social conditions (e.g., stigmatization of homelessness, drug use, gender-based violence) [12]. Mitigation of risk factors should lead to reduced drug use and drug-related harms, in part by addressing the complex, mutually reinforcing, relationship between drug use and homelessness [51]. Though the interview protocol did not explicitly ask women to describe their substance use patterns, some reported reduced use by virtue of being inside. The co-directors affirmed that guests reduced their substance use. Considering that homelessness is associated with a 12-fold risk of fatal overdose, engagement with this population at drop-in centers, overdose prevention centers, healthcare settings, or shelters is vital to reduce fatalities [13, 51, 52].

### The impact of shelter staff

Shelter staff were integral to the success of this shelter. Their compassionate and non-judgmental approach to engagement fostered trusting, safe relationships valued by with the guests [49, 52–54]. The shelter’s approach to drug use supported positive relationships between guests and staff, as staff did not have to surveille guests’ substance use [15]. In most cases, the guests and staff expressed shared perspectives on the shelter environment, demonstrating the effectiveness of the shelter’s implementation.

The primary discrepancy was the staff’s expectation that guests would decrease their substance use. The shelter did not require guests to reduce their substance use or participate in addiction treatment. Nevertheless, some shelter staff wished that the guests would engage in treatment or commit to recovery, a position likely influenced by their previous work in treatment settings or personal experiences [52]. This tension points toward the complexities of harm reduction in practice. While staff endorsed policies that encouraged return to the shelter and safer drug use practices, some struggled to embrace the ideological underpinnings of harm reduction (e.g., autonomy, self-determination). Working in a harm reduction setting is challenging, as it may require staff to provide support and care for guests, while setting aside their personal beliefs about substance use, addiction, and recovery. Staff perspectives reflect research on the acceptability of harm reduction for adults experiencing homelessness with SUDs. Providers in other settings have noticed the benefits of harm reduction (e.g., increased honesty, mutual respect with guests) and been conflicted about whether harm reduction prevented them from “doing enough” to help guests [55, 56].

Notably, the participants in this evaluation did not identify “reducing drug use” or “receiving help with drug use” as reasons for entering or staying in the shelter. Even though staff held these motivations on behalf of the guests, none of the guests indicated they felt pressured to reduce their consumption or experienced substance-related stigma.

## Limitations

The findings from this study should be considered within the context of the following limitations. Prior to the interviews, the researchers were unknown to the guest participants, and this lack of intimacy may have diminished their willingness to discuss their experiences in depth and could have led to social desirability bias [57]. All but one guest interviewed was living in the shelter at the time of the evaluation, which limited our understanding of why guests decided to leave. Similarly, we interviewed staff who agreed to participate during their working hours, none of whom were critical of the shelter. Learning from staff with a diversity of perspectives is an important next step to identify areas for improvement. Future programming and research should focus on refining this model, involving people who use drugs in program design and evaluation, and further studying its effects on guest and staff well-being.

## Conclusion

In the absence of affordable, permanent, safe housing for all, there is a pressing need to establish and implement pragmatic strategies to mitigate the dangers of unsheltered homelessness. Solutions for unsheltered women, who are subject to disproportionate trauma and violence, are especially critical. Homeless service providers should train and support staff to enact a harm reduction approach in these settings. Although low-threshold harm reduction shelters do not address the structural causes of homelessness, they can provide respite, safety, and dignity for this population. Future low-threshold shelters should focus on empowering people with living experience with unsheltered homelessness and substance use disorder to participate in shelter design and implementation.

### Supplementary Information

**Additional file 1: Fig. S1.** Shelter bedroom.

**Additional file 2: Fig. S2.** Common lounge area.

**Additional file 3: Fig. S3.** Kitchen and dining area.

**Additional file 4: Fig. S4.** Amnesty boxes and guest lockers.

## Data Availability

The datasets used and/or analyzed during the current study are not available to protect the confidentiality of the participants.
