# Supplementary figures and images for: “I don’t think of it as a shelter. I say I’m going home”: a qualitative evaluation of a low-threshold shelter for women who use drugs
Source: Harm Reduct J. 2024 Feb 19;21:44. doi: 10.1186/s12954-024-00930-1 (PMC10877776; doi:10.1186/s12954-024-00930-1)

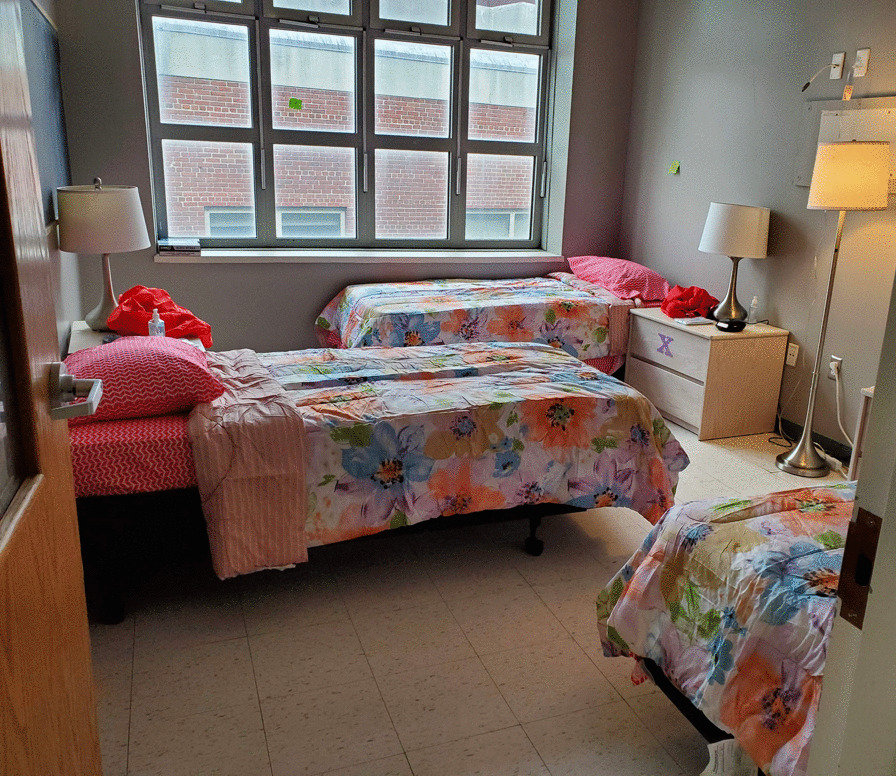

Supplement: Supplementary file 1 — Additional file 1: Fig. S1. Shelter bedroom. [file 12954_2024_930_MOESM1_ESM.jpg]

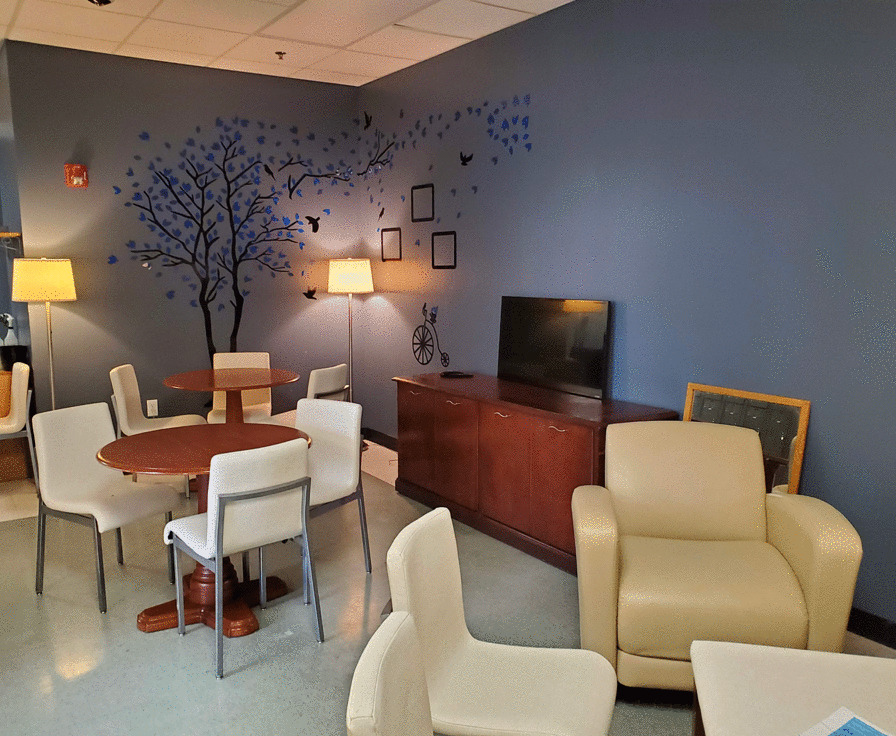

Supplement: Supplementary file 2 — Additional file 2: Fig. S2. Common lounge area. [file 12954_2024_930_MOESM2_ESM.jpg]

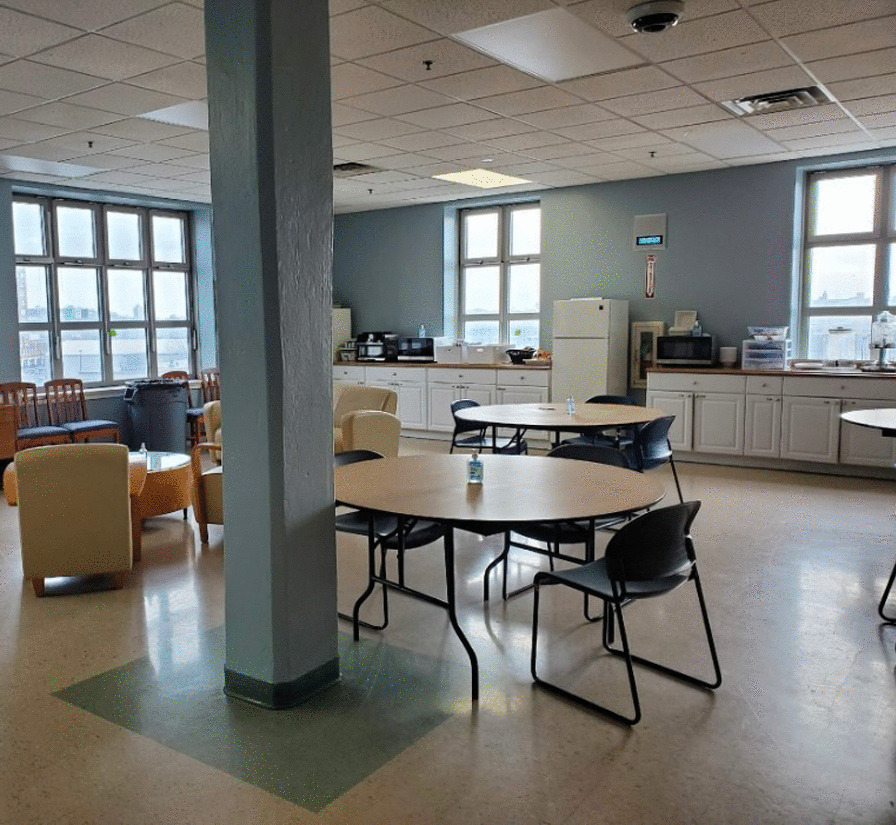

Supplement: Supplementary file 3 — Additional file 3: Fig. S3. Kitchen and dining area. [file 12954_2024_930_MOESM3_ESM.jpg]

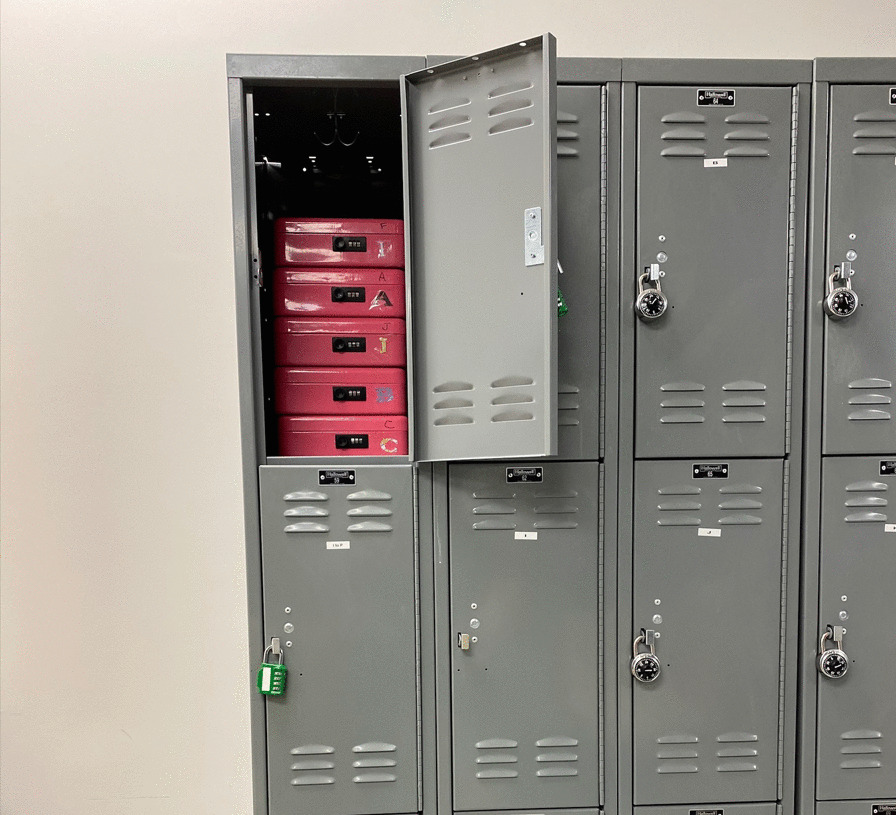

Supplement: Supplementary file 4 — Additional file 4: Fig. S4. Amnesty boxes and guest lockers. [file 12954_2024_930_MOESM4_ESM.jpg]
